# Performance of Donor‐Derived Cell‐Free DNA in Surveillance and For‐Cause Biopsies in Pediatric Kidney Transplant Recipients

**DOI:** 10.1111/petr.70215

**Published:** 2025-10-30

**Authors:** Stella Kilduff, Joseph Fishbein, Carlos Becerril‐Romero, Matthew Switalski, Debora Matossian, Priya S. Verghese

**Affiliations:** ^1^ Ann & Robert H. Lurie Children's Hospital of Chicago Chicago Illinois USA; ^2^ Northwestern University Feinberg School of Medicine Chicago Illinois USA

**Keywords:** donor‐derived cell‐free DNA, kidney transplantation, pediatric, rejection

## Abstract

**Background:**

Donor‐derived cell‐free DNA (dd‐cfDNA), a biomarker demonstrated to increase with allograft injury, has been considered a possible diagnostic tool for allograft rejection in place of the current gold standard which is invasive kidney biopsies. We tested whether dd‐cfDNA levels were predictive of rejection in our single‐center cohort of pediatric kidney transplant (KT) recipients.

**Methods:**

All primary pediatric KT recipients that had a dd‐cfDNA level obtained within a month of any kidney biopsy, either surveillance or for‐cause were included. Descriptive analysis was performed stratified by rejection status. Univariate analysis was performed to assess the association between median dd‐cfDNA levels and rejection by each biopsy time point. dd‐cfDNA levels were then further stratified by rejection type (no rejection, T‐cell mediated, antibody mediated, or mixed). Diagnostic performance metrics of dd‐cfDNA for detecting rejection were evaluated.

**Results:**

Forty pediatric KT recipients had 44 biopsies, 21 (48%) of which demonstrated rejection. Acute cellular, antibody‐mediated, and mixed rejection occurred in 12 (57%), 6 (29%), and 3 (14%) respectively. The median dd‐cfDNA level at the time of biopsy in those with and without rejection was 1.7 (95% CI: 0.2, 3.3) and 0.3 (95% CI: 0.2, 0.7), respectively (*p* = 0.15). dd‐cfDNA levels prior to for‐cause biopsies were significantly higher in patients with rejection (0.3 vs. 2.7; *p* = 0.02). dd‐cfDNA levels at surveillance biopsies did not differ significantly by rejection status or type of rejection. dd‐cfDNA levels ≥ 1 diagnosed rejection with a sensitivity of 52% and specificity of 83%.

**Conclusions:**

Elevated dd‐cfDNA levels were associated with rejection in for‐cause biopsies but not in surveillance biopsies.

## Graphical abstract

Elevated dd‐cfDNA levels were associated with rejection in for‐cause biopsies but not surveillance biopsies in a single‐center pediatric kidney transplant cohort.

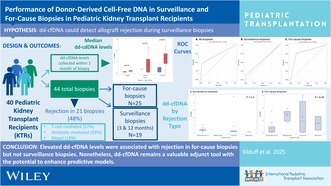

**Abbreviations**
CAKUT: congenital anomalies of the kidney and urinary tract
dd‐cfDNA: donor‐derived cell‐free dna
DSA: donor‐specific antibodies
ESKD: end‐stage kidney disease
HLA: human leukocyte antigens
PRA: peak panel reactive antibody

## 1 Introduction

Kidney biopsies are the gold standard for diagnosing transplant rejection. They are done for cause in kidney recipients with graft dysfunction, and in many centers routinely as surveillance to diagnose subclinical rejection even before the patient has clinical or laboratory markers suggestive of graft injury [1]. Given the invasive nature of biopsies, the development of a biomarker is of great interest to the transplant community. Donor‐derived cell‐free DNA (dd‐cfDNA) is a biomarker that increases with allograft injury and has demonstrated utility in the diagnosis of acute allograft rejection. This has prompted some transplant centers to utilize dd‐cfDNA levels, donor‐specific antibodies (DSA) and serum creatinine trends, to decide when to perform kidney transplant biopsies. Elevations in dd‐cfDNA ≥ 1% are widely considered a reliable marker for detecting acute rejection in adult and pediatric kidney transplant recipients [2, 3, 4, 5, 6, 7, 8]. However, the ability of dd‐cfDNA to predict rejection in surveillance biopsies is less understood. A recent adult study reported that with a 1% dd‐cfDNA cutoff, the sensitivity for detecting rejection in surveillance biopsies was 0%, while the specificity was 89% [9]. In a single‐center pediatric study including 17 surveillance biopsies, only one of the four patients with rejection had a dd‐cfDNA level > 1% [10].

The purpose of this study was to assess whether dd‐cfDNA was predictive of rejection in primary pediatric kidney transplant recipients both for subclinical rejection (at the time of 3‐month and 12‐month posttransplant surveillance biopsies), and when there was clinical evidence of graft dysfunction (at the time of for‐cause kidney transplant biopsy). We further examined dd‐cfDNA levels by type of rejection (no rejection, t‐cell mediated, antibody mediated, or mixed). We hypothesized that increased dd‐cfDNA levels ≥ 1% would be strongly linked to rejection in both surveillance and for‐cause biopsies, and that dd‐cfDNA levels would differ significantly depending on the type of rejection, with higher levels observed in cases of antibody‐mediated rejection (ABMR).

## 2 Patients and Methods

### 2.1 Study Population

We included all pediatric recipients at Ann & Robert H. Lurie Children's Hospital of Chicago who underwent a primary kidney transplant between June 2015 and October 2023 and had a dd‐cfDNA level up to 1 month prior to any kidney biopsy. Data were retrospectively collected to include demographic data such as recipient age, biological sex, and race/ethnicity. Clinical data included the cause of end‐stage kidney disease (ESKD) [categorized as congenital anomalies of the kidney and urinary tract (CAKUT), glomerular disease, cystic disease, or other], body mass index (BMI), peak panel reactive antibody (PRA) (class I or class II), donor type (deceased, living related, or living unrelated), induction (Basiliximab or Anti‐thymocyte globulin) and maintenance immunosuppression therapy (Tacrolimus, Mycophenolate mofetil, Prednisone), de novo human leukocyte antigen (HLA) DSA and BK status. Per center protocol, all recipients of a primary deceased donor allograft who were ≥ 12 years of age at the time of transplant received antithymocyte globulin (1.5 mg/kg/dose ×3 doses) and living donor recipients and/or those < 12 years of age received induction with Basiliximab (10 mg if < 35 kg or 20 mg if ≥ 35 kg, ×2 doses). This study was approved by the institutional review board (IRB) for Ann & Robert H. Lurie Children's Hospital of Chicago (IRB number: 2024‐6889).

### 2.2 Outcome Measurement

The primary outcome of interest was the median dd‐cfDNA level, measured using commercially available AlloSure test kits. Blood samples were collected via venipuncture into Streck tubes, then shipped and analyzed by a Clinical Laboratory Improvement Amendments (CLIA)‐certified laboratory at CareDx Inc. in Brisbane, CA. dd‐cfDNA levels were obtained per center protocol every 3 months starting at 3 months post‐transplant and every 6 months when > 27 months post‐transplant, with additional for‐cause testing performed in response to changes in serum creatinine or other clinical concerns. Kidney biopsies were classified as either surveillance biopsies, which were performed per center protocol at 3‐ and 12‐month post‐transplant, or for‐cause biopsies. For‐cause biopsy indications included increased serum creatinine alone in 17 of 25 biopsies, a combination of elevated creatinine and dd‐cfDNA level in one case, elevated creatinine with BK viremia in one case, de novo DSA alone in two cases, and a combination of de novo DSA and elevated dd‐cfDNA level in four cases. The closest measurement of serum creatinine and DSA within 1 month prior to biopsy was also collected. Biopsies underwent Banff classification by an independent pathologist and were classified as T‐cell mediated rejection (TCMR), ABMR, or mixed rejection [11]. Borderline rejection was grouped with TCMR due to overlapping histologic features, although the clinical management differs at our center. Borderline rejection is treated with oral steroids, while Banff 1A and higher grades receive intravenous pulse steroids followed by a taper, with thymoglobulin added for cases above Banff 1A.

### 2.3 Statistical Analysis

Descriptive analysis of the study cohort was performed stratified by rejection status. Associations of dd‐cfDNA and rejection status were first analyzed as a continuous variable for each biopsy time point (3 months surveillance biopsy, 12 months surveillance, and for‐cause biopsy). dd‐cfDNA levels were subsequently analyzed for both surveillance and for‐cause biopsies and the type of rejection (no rejection, TCMR, ABMR, or mixed). We evaluated associations between a dd‐cfDNA cutoff of ≥ 1% and biopsy indication, rejection type, and presence of DSA. Diagnostic performance metrics including receiver operating characteristic (ROC) curves, sensitivity, specificity, positive predictive value (PPV), and negative predictive value (NPV) were calculated for detecting rejection overall and stratified by surveillance versus for‐cause biopsies. Diagnostic performance was also assessed by combining DSA status with dd‐cfDNA levels. Additionally, the optimal dd‐cfDNA cutoff value was determined separately for all biopsies, surveillance biopsies, and for‐cause biopsies by identifying the point with the highest Youden's index value. Statistical analysis was performed using SAS version 7.15.

## 3 Results

There were 40 sequential primary pediatric kidney recipients included in the study (Table 1). Two recipients underwent two biopsies each during the study period, so clinical data is reported for 40 recipients, while a total of 44 biopsies were reviewed. The cohort was predominantly male (58%) with a median age at the time of transplant of 12.3 years (95% confidence interval [CI]: 5.7, 14.5). Most recipients received kidney transplants from deceased donors (55%), were treated with Basiliximab induction (65%), and were maintained on a regimen of tacrolimus (97.5%) and mycophenolate mofetil (95%). Biopsy‐proven rejection was significantly associated with younger age at transplant (11.2 vs. 13.3 years; *p* = 0.02) but was not significantly associated with any other patient characteristics. BK viremia was present in six out of the 44 biopsies which included five recipients with BK viremia and one with BK nephropathy on biopsy. Median dd‐cfDNA levels were higher in recipients with BK compared to those without [2.6 (95% CI: 1.4, 3.5) vs. 0.33 (0.2, 1.1)] (Table S1). Rejection was seen in two recipients with BK viremia, one with ABMR, and one with mixed rejection.

**TABLE 1:** Demographics and clinical data of pediatric kidney transplant recipients by rejection status.

Characteristics	Overall (*n* = 40)	No rejection (*n* = 23)	Rejection (*n* = 19)	*p*
Age at transplant (years)	12.3 (5.7, 14.5)	13.3 (10, 16.7)	11.2 (3.2, 12.7)	**0.02**
Male	23 (57.5%)	13 (56.5%)	10 (52.6%)	0.55
Race				
Caucasian	11 (27.5%)	7 (30.4%)	5 (26.3%)	0.99
African‐American	7 (17.5%)	4 (17.4%)	3 (15.8%)	
Other	22 (55.0%)	12 (52.2%)	11 (57.9%)	
Hispanic or Latino	19 (47.5%)	11 (47.8%)	9 (47.4%)	0.99
BMI at transplant				
Underweight: < 18.5	22 (55.0%)	11 (47.8%)	13 (68.4%)	0.14
Healthy: 18.5–24.9	13 (32.5%)	7 (30.4%)	6 (31.6%)	
Overweight: 25–29.9	3 (7.5%)	3 (13.0%)	0 (0%)	
Obese: > 30	2 (5.0%)	2 (8.7%)	0 (0%)	
Cause of ESKD				
CAKUT	15 (37.5%)	9 (39.1%)	6 (31.6%)	0.49
Glomerular disease	17 (42.5%)	11 (47.8%)	7 (36.8%)	
Cystic disease	2 (5.0%)	1 (4.3%)	1 (5.3%)	
Other	6 (15.0%)	2 (8.7%)	5 (26.3%)	
Peak panel reactive antibody (PRA)				
HLA Class I	0 (0, 1.5)	0 (0, 4)	0 (0, 0)	0.71
HLA Class II	0 (0, 0)	0 (0, 0)	0 (0, 0)	0.57
Donor type				
Deceased	22 (55.0%)	12 (52.2%)	11 (57.9%)	0.99
Living related	13 (32.5%)	8 (34.8%)	6 (31.6%)	
Living unrelated	5 (12.5%)	3 (13.0%)	2 (10.5%)	
Induction therapy				
Basiliximab	26 (65.0%)	15 (65.2%)	13 (68.4%)	0.83
Antithymocyte globulin	14 (35.0%)	8 (34.8%)	6 (31.6%)	
Maintenance immunosuppression				
Tacrolimus	39 (97.5%)	23 (100%)	18 (94.7%)	—
Mycophenolate mofetil	38 (95.0%)	23 (100%)	17 (89.5%)	
Prednisone	9 (22.5%)	7 (30.4%)	2 (10.5%)	

Biopsy and clinical data	Overall	No rejection	Rejection	*p*
	(*n* = 44)	(*n* = 23)	(*n* = 21)	
Reason for biopsy				
3‐months surveillance	14 (31.8%)	10 (43.5%)	4 (19%)	0.11
12‐months surveillance	5 (11.4%)	1 (4.3%)	4 (19%)	
For‐cause biopsy^a^	25 (56.8%)	12 (52.2%)	13 (62%)	
DSA < 1 month prior to biopsy				
Yes	13 (29.5%)	4 (17.4%)	9 (42.9%)	0.09
No	31 (70.5%)	19 (82.6%)	12 (57.1%)	
BK viremia	6 (13.6%)	4 (17.4%)	2 (9.5%)	1.0
Serum creatinine prior to biopsy (mg/dL)	0.9 (0.8, 1.2)	1.0 (0.8, 1.3)	0.9 (0.5, 1.0)	**0.04**
dd‐cfDNA level prior to biopsy	0.4 (0.2, 1.8)	0.3 (0.2, 0.7)	1.7 (0.2, 3.3)	0.15
Timing of dd‐cfDNA (days prior to biopsy)	11 (5.5, 17.5)	11 (4, 16)	10 (6, 19)	0.7

*Note:* Frequencies are presented as *n* (%) and continuous variables as median (interquartile range). Bold values denote statistical significance at the *p* < 0.05 level.

Abbreviations: BMI, body mass index; dd‐cfDNA, donor‐derived cell‐free DNA; DSA, donor‐specific antibodies; ESKD, end‐stage kidney disease; HLA, human leukocyte antigen.

^a^ For‐cause biopsies include those with an increase in serum creatinine, dd‐cfDNA level, or new DSA.

Rejection was diagnosed in 21 of the 44 biopsies, with acute cellular, antibody‐mediated, and mixed rejection occurring in 57%, 29%, and 14% of cases, respectively. Borderline rejection made up 58% of all cellular rejection cases; 57.5%, 32.5%, and 10% were for‐cause biopsies, 3‐month, and 12‐month surveillance biopsies, respectively. Although not statistically significant, the median dd‐cfDNA level at the time of biopsy in those with rejection was 1.7 (95% CI: 0.2, 3.3) versus 0.3 (95% CI: 0.2, 0.7) in those without rejection (*p* = 0.15). The median time of dd‐cfDNA levels to biopsy was 11 days. Among the TCMR cohort, median dd‐cfDNA levels were higher in Banff 1A and greater rejection cases compared to borderline rejection cases; however this difference was not statistically significant [0.2 (95% CI: 0.19, 0.33) vs. 1.2 (95% CI: 0.15, 4.95)].

### 3.1 dd‐cfDNA in Surveillance Versus For‐Cause Biopsies

**FIGURE 1:**
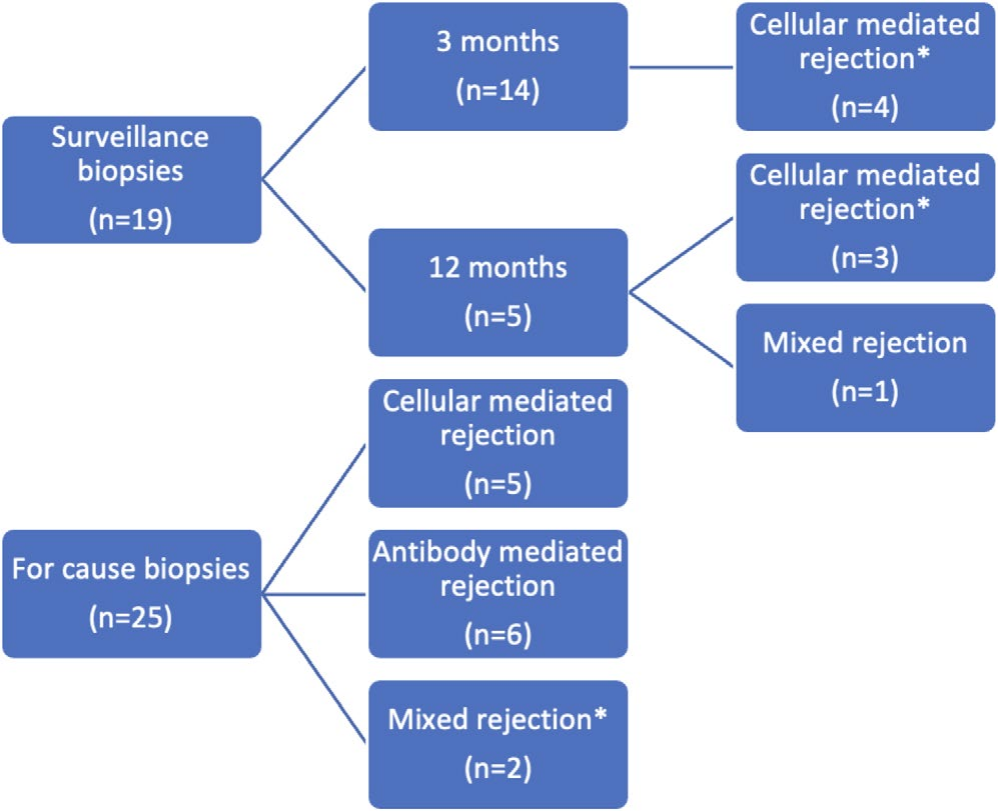
Reason for biopsy categorized by rejection types of the 44 allograft biopsies. *Borderline rejection: 3 months *n* = 4; 12 months *n* = 2; For‐cause: *n* = 1.

**FIGURE 2:**
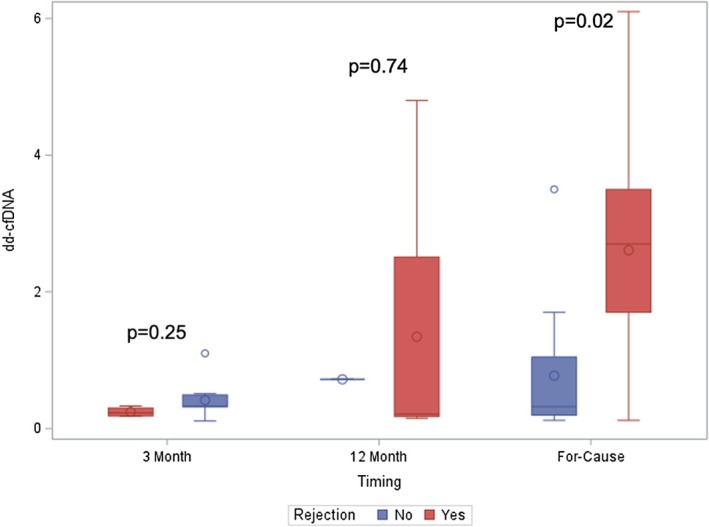
The association of dd‐cfDNA at the timing of assessment with rejection status. dd‐cfDNA values at surveillance (3 months and 12 months) and for‐cause biopsies.

**FIGURE 3:**
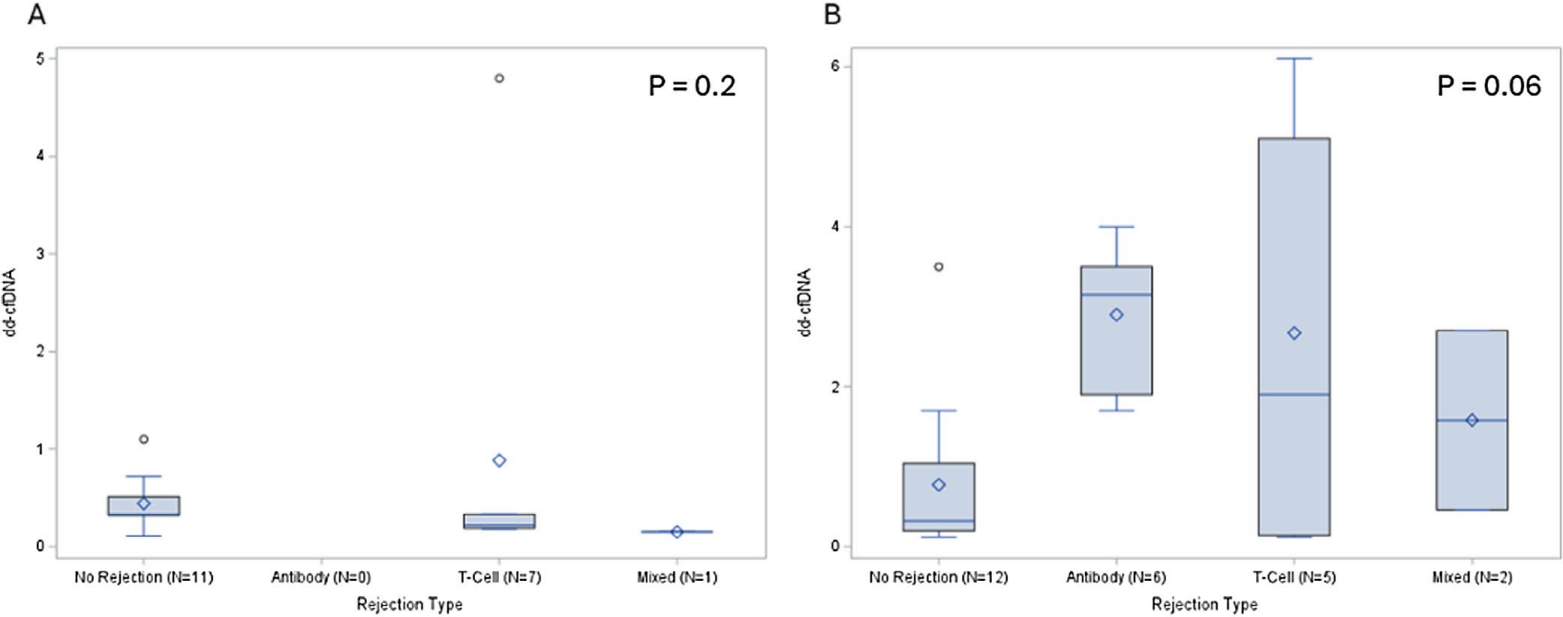
dd‐cfDNA by rejection type for surveillance versus for‐cause biopsies. dd‐cfDNA stratified by rejection type: No rejection, antibody‐mediated, t‐cell mediated and mixed rejection for (A) surveillance biopsies (B) for‐cause biopsies.

Of the 19 surveillance biopsies, 42% showed rejection, with the majority being borderline cellular‐mediated rejection and none showed ABMR alone (Figure 1). In the 25 for‐cause biopsies, six recipients had ABMR, five had TCMR, and two had mixed rejection. A higher proportion of biopsy‐proven rejections was observed in the 12‐month surveillance (four out of five biopsies, 80%) and for‐cause biopsies (13 of 25, 52%), compared to the 3‐month surveillance biopsies, where only four out of 14 cases (29%) had rejection. dd‐cfDNA levels at surveillance biopsies (3 and/or 12 months) did not differ significantly by rejection status (Figure 2). However, median dd‐cfDNA levels prior to for‐cause biopsies were significantly higher in patients with rejection (0.3 vs. 2.7; *p* = 0.02). dd‐cfDNA levels did not significantly differ by rejection type for either surveillance or for‐cause biopsies (Figure 3).

### 3.2 Diagnostic Performance of dd‐cfDNA Cutoffs

Among all biopsies, dd‐cfDNA levels ≥ 1% were significantly associated with an increased likelihood of rejection (Table 2). Rejection was observed in 73% (11/15) of biopsies with dd‐cfDNA ≥ 1% compared to 34% (10/29) with dd‐cfDNA < 1% (*p* = 0.03). The four biopsies without rejection that had dd‐cfDNA levels ≥ 1% included one case with mild interstitial fibrosis and tubular atrophy (IFTA), one case of BK nephropathy, one case with mild IFTA and focal mild arteriolar hyalinosis, and one case with nephrocalcinosis. When stratified by reason for biopsy, dd‐cfDNA levels ≥ 1% were significantly associated with for‐cause biopsies (77% vs. 25%; *p* = 0.02), but not with surveillance biopsies (50% vs. 41%; *p* = 1.0). Among rejection cases, ABMR was observed exclusively in the dd‐cfDNA ≥ 1% group (6/11), while TCMR and mixed rejection were more common in the dd‐cfDNA < 1% group (10 total: 8 cellular, 2 mixed). Among recipients with DSA, a larger proportion had dd‐cfDNA levels ≥ 1%, compared to those with dd‐cfDNA < 1%.

**TABLE 2:** Association of clinical factors with dd‐cfDNA ≥ 1% versus < 1% with rejection in pediatric kidney transplant recipients.

	dd‐cfDNA ≥ 1%	dd‐cfDNA < 1%	*p*
Rejection on all biopsies	11/15 (73%)	10/29 (34%)	**0.03**
Rejection on surveillance biopsies	1/2 (50%)	7/17 (41%)	1.0
Rejection on for‐cause biopsies	10/13 (77%)	3/12 (25%)	**0.02**
Rejection type			
Cellular mediated	4/11 (36%)	8/10 (80%)	**0.02**
Antibody mediated	6/11 (55%)	0	
Mixed	1/11 (9%)	2/10 (20%)	
DSA	7/15 (47%)	6/29 (21%)	0.07

*Note:* Frequencies are presented as *n* (%). Bold values denote statistical significance at the *p* < 0.05 level.

Abbreviations: dd‐cfDNA, donor‐derived cell‐free DNA; DSA, donor‐specific antibodies.

**FIGURE 4:**
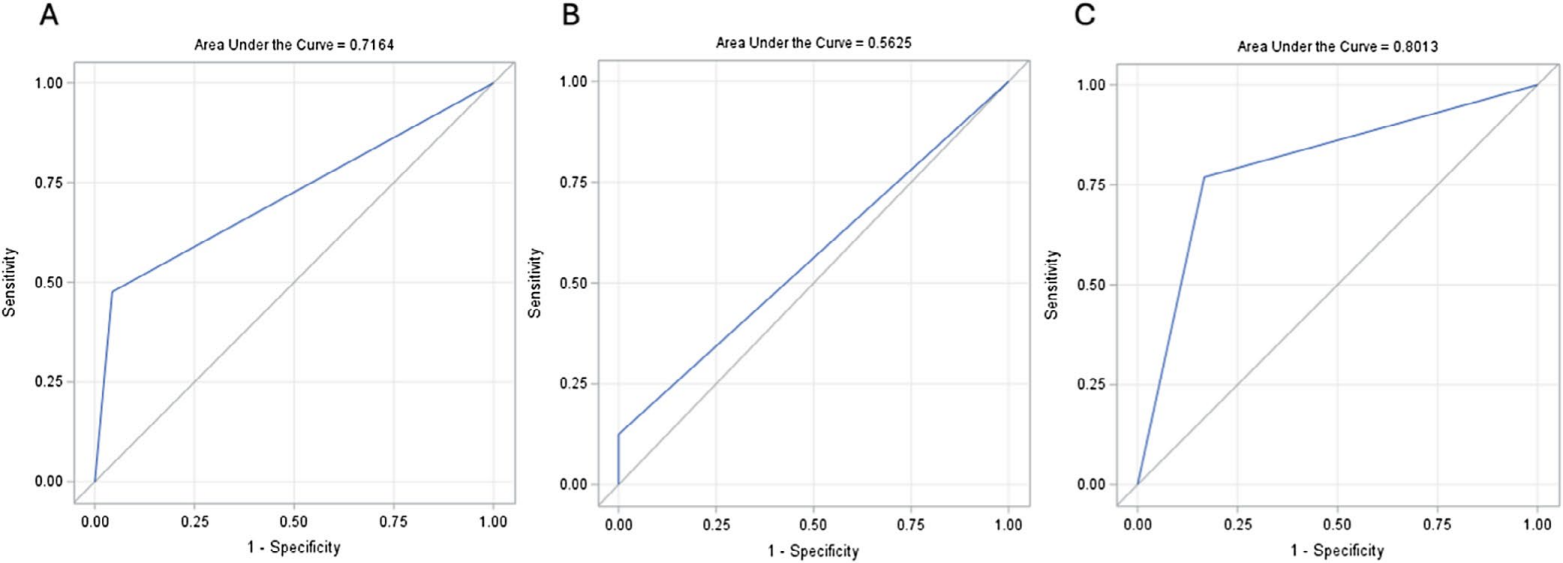
ROC curve analysis of dd‐cfDNA ≥ 1% cutoff in all biopsies, surveillance biopsies and for‐cause biopsies. Receiver operating characteristic (ROC) curve analysis of dd‐cfDNA for predicting rejection in (A) all biopsies, (B) surveillance biopsies, and (C) for‐cause biopsies.

The area under the curve (AUC) demonstrated good discriminative ability for a dd‐cfDNA cutoff test of ≥ 1% in predicting rejection among for‐cause biopsies, with an area under the curve of 0.80 (Figure 4C). In contrast, the AUC was 0.56 for surveillance biopsies (Figure 4B). When considering all biopsies, the AUC was 0.72 (Figure 4A). Using a dd‐cfDNA cutoff test of ≥ 1% demonstrated an overall sensitivity of 52% and specificity of 83% for detecting allograft rejection in pediatric kidney transplant recipients, with a PPV of 61% and NPV of 93% (Table 3). For‐cause biopsies demonstrated improved diagnostic performance, with both sensitivity and PPV reaching 77% supporting its utility in clinically indicated evaluations. In contrast, among surveillance biopsies, dd‐cfDNA ≥ 1% had markedly lower sensitivity (13%) and NPV (59%), though specificity remained high (91%) with a PPV of 50%.

**TABLE 3:** Diagnostic performance of dd‐cfDNA cutoffs for detecting rejection in pediatric kidney transplant recipients.

	All biopsies	Surveillance biopsies	For‐cause biopsies
dd‐cfDNA cutoff ≥ 1%	*n* = 44	*n* = 25	*n* = 19
Sensitivity	52%	13%	77%
Specificity	83%	91%	75%
Positive predictive value	73%	50%	77%
Negative predictive value	66%	59%	75%
dd‐cfDNA cutoff ≥ 1%+DSA+	*n* = 13	*n* = 11	*n* = 2
Sensitivity	67%	75%	0%
Specificity	75%	67%	100%
Positive predictive value	86%	86%	.
Negative predictive value	50%	50%	50%
Optimal dd‐cfDNA cutoffs	1.7	4.8	1.9
Sensitivity	52.4%	12.5%	69.2%
Specificity	91.3%	100%	91.7%
Positive predictive value	84.6%	100%	90%
Negative predictive value	67.7%	61.1%	73.3%

Combining DSA status with the dd‐cfDNA ≥ 1% improved diagnostic performance. The sensitivity was increased to 67% among all biopsies and to 75% among surveillance biopsies. Based on Youden's index, the best operating point corresponded to a dd‐cfDNA cutoff of 1.7% for all biopsies. At this threshold, the test achieved a sensitivity of 52.4% and a specificity of 91.3%. The optimal cutoff values for surveillance biopsies and for‐cause biopsies were 2.8% and 1.9%, respectively.

## 4 Discussion

In our single center study of pediatric primary kidney transplant recipients, dd‐cfDNA levels were not predictive of rejection in recipients who underwent surveillance biopsies. However, they did appear to predict rejection at for‐cause biopsies, most commonly when the recipients had ABMR. While biopsies with rejection had a higher median dd‐cfDNA (1.7) than those without rejection (0.3), this difference did not achieve statistical significance, presumably due to limited sample size. The dd‐cfDNA cutoff of ≥ 1% showed a significant association with rejection in both the overall biopsy cohort and for‐cause biopsies, consistent with the previously reported studies [2, 7]. However, this association was not significant in surveillance biopsies.

Unique to our study, dd‐cfDNA levels were assessed at defined biopsy time points and stratified the analysis by rejection type, offering a more detailed and clinically meaningful evaluation. We observed higher rejection rates during the 12‐month surveillance biopsies compared to the 3‐month surveillance biopsies; however, median dd‐cfDNA levels did not significantly differ between those with or without rejection. Although we did not include dd‐cfDNA values from patients without biopsies, the levels observed in patients without rejection (Tables 1 and 2) provide a practical reference for expected dd‐cfDNA levels in stable patients within our cohort.

Prior studies have shown that dd‐cfDNA correlates more strongly with antibody‐mediated rejection [2] and higher grades of cellular rejection (Banff > 1A) [12]. A more recent study of 196 pediatric kidney transplant recipients reported dd‐cfDNA demonstrated good discriminatory ability, with AUC values of 0.73 (95% CI: 0.63–0.84) for detecting TCMR and 0.79 (95% CI: 0.70–0.89) for detecting ABMR [13]. In contrast, our study did not find a significant difference in median dd‐cfDNA levels across rejection types, despite nearly half of the biopsies being antibody‐mediated, including three with mixed rejection. This discrepancy may be due to the relatively high proportion of borderline rejection cases in our surveillance biopsies, which are known to induce only mild histologic changes and elicit lower dd‐cfDNA elevations compared to Banff 1A and greater TCMR. Nonetheless, recognizing borderline rejection remains crucial, as untreated borderline changes have been associated with increased incidence of subsequent TCMR, progression to chronic allograft injury, and formation of de‐novo DSA [14, 15]. For this reason, these cases were included in our TCMR cohort. Additionally, the limited sample size and the predominance of ABMR cases in the for‐cause biopsy group may have limited our ability to detect statistically significant differences. Notably, ABMR was significantly more frequent among recipients with dd‐cfDNA levels ≥ 1%. We also cannot exclude the influence of chronic changes or subclinical injury not reflected by dd‐cfDNA levels, which could further obscure clear correlations.

Diagnostic performance was higher in for‐cause biopsies compared to surveillance biopsies as reflected by increased AUC, sensitivity, and PPV, indicating stronger predictive utility in patients undergoing biopsy due to clinical suspicion. Given the higher prevalence of rejection in for‐cause biopsies compared to surveillance biopsies, predictive values such as PPV and NPV naturally differ between these clinical settings, influencing the interpretation and utility of dd‐cfDNA measurements. In contrast, surveillance biopsies had lower PPV, reflecting more false positives, and a reduced NPV, indicating that a negative dd‐cfDNA result did not reliably exclude the possibility of underlying rejection. The lower PPV may in part reflect the higher proportion of borderline rejection diagnoses in our surveillance cohort. The predictive power of dd‐cfDNA was largely driven by its strong discriminatory performance in for‐cause biopsies (AUC 0.80), which increased the overall AUC to 0.72, in contrast to the poor performance observed in surveillance biopsies (AUC 0.56). This discrepancy is further demonstrated by the high optimal dd‐cfDNA cutoff point identified in surveillance biopsies, which was 4.8%. These findings indicate that while dd‐cfDNA levels ≥ 1% serve as a useful non‐invasive tool for ruling out rejection in clinically indicated scenarios, its utility in detecting subclinical rejection during routine surveillance is limited when used as a standalone biomarker. This is consistent with findings from Huang et al., who reported that most clinically stable patients with elevated dd‐cfDNA did not experience rejection or graft dysfunction during follow‐up, highlighting the need for cautious interpretation of elevated levels in low‐risk settings [16]. The addition of DSA status did improve predictability in our cohort. Building on similar observations, Hogan et al. demonstrated that combining dd‐cfDNA with clinical variables such as recipient age, time since transplant, history of rejection, estimated glomerular filtration rate, proteinuria, and DSA status enhanced diagnostic performance, yielding an AUC of 0.84 (95% CI: 0.79–0.90) across all biopsies and 0.73 (95% CI: 0.67–0.79) in a sensitivity analysis limited to surveillance biopsies [13]. A recent study by Bromberg et al. reported that using a combination of dd‐cfDNA threshold ≥ 1% and a relative change value (RCV) of ≥ 61% increase from the prior level, even if below 1%, improved the overall accuracy of diagnosing biopsy proven rejection [17]. While our study utilized fractional dd‐cfDNA as a percentage of total cfDNA, emerging evidence suggests that absolute dd‐cfDNA concentrations may also improve diagnostic accuracy by accounting for variability in total cfDNA levels [18]. Other promising biomarkers such as urinary chemokines (CXCL9 and CXCL10) and Molecular Microscope Diagnostic System (MMDx) may further complement dd‐cfDNA in detecting rejection [19, 20, 21].

The limitations of our study include its retrospective design and single‐center setting, which resulted in a relatively small sample size that may have reduced the ability to detect statistically significant differences. Variability among surveillance biopsies was limited, as none demonstrated isolated ABMR. Furthermore, although dd‐cfDNA levels were collected within 1 month prior to biopsy, variation in timing may have influenced the interpretation of temporal relationships, as earlier or transient rises in dd‐cfDNA may have been missed. A previous study found that dd‐cfDNA elevations occurred 5 months before ABMR and 2 months before TCMR compared to recipients without rejection [22]. It is also worth noting that recipients with BK viremia and one with BK nephropathy were not excluded from the present analysis, despite prior studies reporting a significant correlation between BK viral load and dd‐cfDNA [4, 13, 23, 24]. This approach represents both a strength and a potential limitation of our study. While including these cases enhances the generalizability and clinical relevance of our findings by simulating real‐world conditions, it may also introduce confounding effects that could influence dd‐cfDNA levels independently of rejection.

In conclusion, our study emphasizes the limitation of dd‐cfDNA as a noninvasive biomarker for rejection in surveillance settings. While dd‐cfDNA demonstrated potential in identifying rejection among clinically indicated biopsies, it lacked sufficient sensitivity to reliably detect subclinical rejection, which was only identified through routine surveillance biopsies. Therefore, dd‐cfDNA alone does not currently support the elimination of surveillance biopsies. In addition, the diagnosis of borderline rejection remains a challenge for dd‐cfDNA‐based monitoring. Nonetheless, dd‐cfDNA remains a valuable adjunct tool with the potential to enhance predictive models when integrated with clinical and laboratory parameters. Prospective, multicenter pediatric studies are warranted to optimize comprehensive, risk‐based prediction frameworks for kidney transplant monitoring.

## Ethics Statement

The study was approved by the institutional review board for Ann & Robert H. Lurie Children's Hospital of Chicago.

## Consent

The need for informed consent was waived due to the use of de‐identified data.

## Conflicts of Interest

Authors Stella Kilduff, Joseph Fishbein, Carlos Becerril‐Romero, Matthew Switalski, and Debora Matossian declare that they have no conflicts of interest or disclosures. Priya S. Verghese is a consultant for Boehringer‐Ingelheim.

## Supporting information


**Table S1:** petr70215‐sup‐0001‐TableS1.docx.

## Data Availability

The data that support the findings of this study are available on request from the corresponding author. The data are not publicly available due to privacy or ethical restrictions.
