# Supplementary material for: Performance of Donor‐Derived Cell‐Free DNA in Surveillance and For‐Cause Biopsies in Pediatric Kidney Transplant Recipients
Source: Pediatr Transplant. 2025 Oct 30;29(8):e70215. doi: 10.1111/petr.70215 (PMC12575418; doi:10.1111/petr.70215)
Supplement: Supplementary file 1 — Table S1: petr70215‐sup‐0001‐TableS1.docx. [file PETR-29-e70215-s001.docx]

**Supplemental Table**

**Table 1. Clinical data on recipients with and without BK viremia**

|  | BK Viremia  (n=6) | No BK Viremia  (n=38) |
| --- | --- | --- |
| Median dd-cfDNA | 2.6 (1.4, 3.5) | 0.33 (0.2, 1.1) |
| Rejection | 2 (33.3%) | 19 (50%) |
| Biopsy Type  For-cause  Surveillance | 4 (66.7%)  2 (33.3%) | 21 (55.3%)  17 (44.7%) |
| Indication for Biopsy  Elevated Creatinine  DSA  Elevated dd-cfDNA  Other* | 4 (66.7%)  0 (0%)  0 (0%)  1 (25%) | 15 (71.4%)  6 (28.6%)  5 (23.8%)  0 (0%) |
| Rejection Type  Antibody mediated  Cellular mediated  Mixed  None | 1 (16.6%)  1 (16.6%)  0 (0 %)  4 (66.8%) | 5 (13.2%)  11 (28.9%)  3 (7.9%)  19 (50%) |

Frequencies are presented as n(%) and continuous variables as median (Interquartile range)

*Other: BK viremia
